# Risk Factors and Treatment Strategy for Retinal Vascular Occlusive Diseases

**DOI:** 10.3390/jcm11216340

**Published:** 2022-10-27

**Authors:** Ryo Terao, Ryosuke Fujino, Tazbir Ahmed

**Affiliations:** 1 Department of Ophthalmology, Graduate School of Medicine, The University of Tokyo, Tokyo 113-8654, Japan; 2 Department of Ophthalmology & Visual Sciences, Washington University School of Medicine in St. Louis, St. Louis, MO 63110, USA

**Keywords:** retinal artery occlusion, retinal vein occlusion, anti-vascular endothelial growth factor, macular edema

## Abstract

Retinal occlusive diseases are common diseases that can lead to visual impairment. Retinal artery occlusion and retinal vein occlusion are included in the clinical entity, but they have quite different pathophysiologies. Retinal artery occlusion is an emergent eye disorder. Retinal artery occlusion is mainly caused by thromboembolism, which frequently occurs in conjunction with life-threatening stroke and cardiovascular diseases. Therefore, prompt examinations and interventions for systemic vascular diseases are often necessary for these patients. Retinal vein occlusion is characterized by retinal hemorrhage and ischemia, which may impair visual function via several complications such as macular edema, macular ischemia, vitreous hemorrhage, and neovascular glaucoma. Even though anti-vascular endothelial growth factor therapy is the current established first-line of treatment for retinal vein occlusion, several clinical studies have been performed to identify better treatment protocols and new therapeutic options. In this review, we summarize the current findings and advances in knowledge regarding retinal occlusive diseases, particularly focusing on recent studies, in order to provide an update for a better understanding of its pathogenesis.

## 1. Introduction

Retinal occlusive diseases are common diseases that cause visual dysfunction and atherosclerosis and thromboembolism partially underlie their pathology [1]. They are categorized into two types of diseases: retinal artery occlusion and retinal vein occlusion. Retinal artery occlusion is an acute eye stroke, caused by acute blockage in the eye, which leads to the sudden and severe loss of vision. Retinal vein occlusion caused by arteriosclerosis and thromboembolism is characterized by retinal hemorrhage, retinal edema, macular ischemia, cotton-wool spots, and tortuous vessels [2,3]. Retinal neovascularization secondary to chronic retinal ischemia may lead to vitreous hemorrhage, retinal detachment, and neovascular glaucoma [4]. There are many differences in the pathology, manifestations, and visual prognosis between retinal artery occlusion and retinal vein occlusion [5]. Therefore, therapeutic approaches to these disorders are entirely different. In this review, we summarized the recent findings regarding the risk factors and treatment strategies for retinal artery occlusion and retinal vein occlusion and discuss their respective perspectives.

## 2. Retinal Artery Occlusion

### 2.1. Etiology of Retinal Artery Occlusion

Retinal artery occlusion includes central retinal arterial occlusion (CRAO), branch retinal arterial occlusion (BRAO), vascular transient monocular vision loss (TMVL), and ophthalmic arterial occlusion (OAO) [5,6]. Among retinal artery occlusions, CRAO and OAO are particularly emergent disorders. Retinal artery occlusion immediately causes retinal infarction, which leads to acute, severe vision loss and/or visual field loss. The inner layer of the retina is supplied by the retinal artery and the outer layer is supplied by choroidal circulation. In CRAO, the loss of arterial inflow to the retina rapidly induces painless, sudden, and unilateral vision loss [7]. Retinal embolus are made of cholesterol (74%), calcified material (10.5%), and platelet-fibrin (15.5%) [8,9]. The retina can be repaired from ischemia lasting less than 100 min, while longer ischemia leads to irreversible and unrepairable deterioration [10]. In the acute phase, the ischemic retina exhibits an edematous change in the inner layer, showing retinal whitening. As the fovea does not have an inner layer, a fovea surrounded by a whitish retina is called a “cherry red spot”, a characteristic typical of CRAO [11]. In recent years, the incident rate of CRAO has been 1.80 per 100,000 persons per year [12]. The incidence increases with age and the incidence among those 80–84 years old is 10.08 per 100,000 persons per year. Males have a higher incidence than females [12,13]. The visual prognosis for CRAO is devastating, 93.2% of which is counting fingers or worse [14]. Sustained arterial occlusion leads to persistent and irreversible vision loss [14,15]. Visual acuity at the initial visit correlates with better visual prognosis [16]. Approximately 30% of people have a cilioretinal artery [17,18], which can supply macula during retinal artery occlusion. Patients with cilioretinal artery sparing generally have a good prognosis [19]. An initial visual acuity in 20% of CRAOs with cilioretinal artery sparing is 20/40 or better [14]. BRAO is the embolic occlusion of the branching artery. The visual prognosis for BRAO is quite different depending on the occlusion site; that is, whether the lesion involves the fovea. An initial visual acuity of approximately 40–50% is 20/40 or better and 80% improve to 20/40 or better at follow-up [20,21]. Here, 20% of BRAO had central scotoma, 13% had inferior central visual field defect, and 53% had peripheral nasal defect [14].

TMVL describes episodes of temporary acute vision loss in one eye and vascular disorders may underlie the etiology. Retinal migraine and retinal vasospasm are relatively benign causes of transient monocular vision loss [22]. Meanwhile, vascular TMVL may be an emergent disease caused by life-threatening vascular events such as carotid occlusion and thromboembolism [23,24]. Amaurosis fugax is one of the most common causes of TMVL, which is an acute monocular vision loss caused by a thromboembolic vascular etiology [25]. The incidence of amaurosis fugax is approximately 14 per 100,000 persons per year [26]. Pathologically, it is caused by a reduced flow of blood to the brain, whole eye, optic nerve, or retina [27,28]. As patients show no ophthalmological abnormalities when they have no symptoms, it is essential for clinicians to take the medical history of patients with TMVL carefully. Recent studies have found orbital ultrasounds to be useful in distinguishing benign transient monocular vision loss and vascular TMVL as well as CRAO by evaluating ophthalmic and central retinal arteries [29,30].

OAO is a vascular obstruction in the ophthalmic artery. ORO is rare, but it may occur iatrogenically by intravascular embolism through blood flow or orbital compartment syndrome [31,32]. OAO has a relatively worse prognosis than other subtypes of retinal artery occlusion, because it is caused by a larger embolus or thrombus than CRAO, which makes it more difficult to dislodge.

### 2.2. Risk Factors for Retinal Artery Occlusion

As described above, there are several subtypes of retinal artery occlusion. However, the underlying etiology and pathology are similar. As these disorders are highly associated with emergent vascular events, the most significance in a strategic approach toward retinal artery occlusion is prompt systemic examinations and triage to identify the presence and/or risk of cardiovascular events [33,34,35,36]. As medical professionals, ophthalmologists are supposed to determine the underlying condition of the retinal artery occlusion as well as provide treatment for acute vision loss. Previously, there have been numerous studies to determine the potential risk of vascular events [5,37,38,39].

Because an embolus can cause retinal artery occlusion, a systemic examination is particularly focused on the risk of mortal stroke and cardiovascular events. CRAO is reportedly associated with migraine, aura, obesity, hypertension, hypercholesterolemia, diabetes, and atrial fibrillation [5,40,41,42]. Young patients with CRAO significantly have several systemic factors, such as a hypercoagulable state, cardiac valvular disease, and migraine, reported to be associated with CRAO [43,44]. A recent study found additional risk factors, including systemic vasculitis, syphilis, and glaucoma, as well as hypertension and carotid stenosis, among young patients [45]. Meanwhile, retinal artery occlusion in aged patients is more likely to be accompanied by acute, serious vascular ischemic events, such as stroke and myocardial infarction, which require emergent surgical treatment interventions [46,47]. For instance, among inpatients with acute CRAO in the United States, 15.3% had stroke and 7.7% had myocardial infarction [46]. Another study, using magnetic resonance and echocardiography, found that 36.7% had critical carotid disease and stroke was found in 37.3% of CRAO inpatients. As a result, 78.6% were diagnosed with new significant disorders. Subclinical atrial fibrillation was found in 15% of CRAO, which is a similar rate to those with cryptogenic stroke [48].

During a two-year follow-up, stroke, myocardial infarction, or death were observed at 32.0%, indicating CRAO as a warning of fatal illness [49]. Other studies found that, at least, a new cardiovascular risk factor was found in approximately 80% of patients with CRAO, and carotid stenosis or cardioembolism were observed in approximately 40% of CRAOs [42,50]. Patients with BRAO have a risk of stroke, myocardial infarction, and all-cause mortality comparable to CRAO [51]. Recent studies investigating the risk factors in CRAO are summarized in Table 1. In turn, 16.9% of patients with acute ischemic stroke had retinal emboli and 9% had acute retinal artery occlusion [52]. Patients with a diagnosis of stroke or coronary heart disease had a higher prevalence of asymptomatic retinal ischemia, indicating the coexistence of cardiovascular diseases and retinal stroke [53].

A systemic evaluation is particularly necessary for patients with CRAO during hospitalization, because the incidence rapidly elevates within 1–2 weeks of the onset of retinal artery occlusion [54,55]. The incidence of stroke in patients with CRAO becomes obviously higher after the occurrence of CRAO than before. The incidence of cardiovascular events was particularly higher within 30 days after the occurrence of CRAO, of which the incidence rate ratio to the incidence of cardiovascular events 180 days prior to CRAO was approximately 14 [56]. Cerebral ischemia was found in 30% of CRAO patients, 25% of BRAO, and 11.8% of TMVL by magnetic resonance imaging (MRI) taken within 7 days after the diagnosis of retinal artery occlusion [57]. Therefore, medical care for some patients with CRAO needs to be shifted toward more emergent and life-threatening diseases. These studies suggest the importance of being alert to the signs of mortal diseases in patients with retinal artery occlusion. As most patients with retinal artery occlusion do not receive examinations necessary for cardiovascular testing [58], healthcare providers must always be aware that the detection of these serious diseases can prevent the occurrence of subsequent stroke and cardiovascular diseases. In fact, patients with retinal artery occlusion have a shorter life expectancy and higher risk of all-cause mortality [59].

**Table 1:** Clinical studies published between 2020 and 2022 that evaluated the association with cardiovascular diseases.

Authors (Year)	Patients (n)	Risk Factors	Reference
Shaikh, I. S., et al. (2020)	RAO with/without stroke (1157, 18,652)	AF (13%), carotid stenosis (43%), coronary artery disease (21%), complicated diabetes (5%), hyperlipidemia (45%), hypertension (67%), and cardiac valvular disease (9%). Cardiac valvular disease, tobacco use, non-stroke cerebrovascular disease, hypertension, and hyperlipidemia were significant risk factors of stroke following RAO.	[60]
Schorr, E. M., et al. (2020)	RAO (4871)	Hypertension (62%), AF (16%), cardiac valvular disease (13%), and heart failure (9%).	[61]
Xiao, Y. Y., et al. (2020)	CRAO (28) and BRAO (17)	Plaques in the carotid artery (89%), MI (7%), history of stroke (40%), smoking (56%). History of stroke was a significant risk factor for RAO.	[62]
Watson, R. A., et al. (2020)	CRAO (64)	AF was detected in 15% by an implantable recorder.	[48]
Vestergaard, N., et al. (2021)	RAO (6628)	Hypertension (28%), AF (11%), and heart failure (9%). The incidence of stroke, MI, and death was significantly higher at follow-up.	[63]
Orskov, M., et al. (2022)	RAO (5683) and stroke (28,415)	Diabetes (15%), arterial hypertension (8%), ischemic heart disease (14%), and peripheral artery disease (9%).	[64]
Kaur, M., et al.(2022)	CVD with RAO (1700)	Diabetes with complications (69%), complicated hypertension (55%), and peripheral vascular diseases (12%).	[65]
Chodnicki, K. D., et al. (2022)	CRAO (89)	Hypertension (92%), hyperlipidemia (53%), diabetes (29%), and history of stroke (32%). In addition, 2.2% developed symptomatic ischemic stroke within 15 days before/after CRAO.	[66]

AF: atrial fibrillation; BRAO: branch retinal artery occlusion; CRAO: central retinal artery occlusion; CVD: cerebrovascular disease; MI: myocardial infarction; RAO: retinal artery occlusion.

Several recent reports have described other risks for retinal vascular occlusion. Vascular dementia may also be associated with retinal artery occlusion. Chan et al. revealed that patients with retinal vascular occlusion had a significantly higher prevalence of cognitive dementia including Alzheimer’s disease, vascular dementia, and dementia with Lewy bodies. However, after adjusting for other significant covariates, there were no significant associations between retinal vascular occlusion and cognitive dementia [67]. Lee et al. found, that among people with ε4 alleles of the apolipoprotein E, those with retinal vascular occlusion had a significantly higher risk of developing vascular dementia at follow-up [68], indicating that retinal vascular occlusion and dementia (particularly vascular dementia) shared an underlying pathogenesis.

Recently, studies have suggested a history of SARS-CoV-2 infection (COVID-19) as a significant risk factor for retinal vascular occlusion. Conjunctival congestion and conjunctivitis are the most common complications of the eye [69]. In addition, as COVID-19 exhibits vascular complications, such as thrombosis [70,71], patients with COVID-19 may suffer from retinal vascular events [72]. The incidence of retinal artery and vein occlusions slightly elevates within 12 weeks after COVID-19. In particular, an episode of COVID-19 within 6 months is a significant high-risk factor for retinal vein occlusion [73,74]. Moreover, COVID-19 exhibits retinal hemorrhage, cotton-wool spots, and dilated retinal arteries and veins [75]. Although more evidence is necessary to elucidate the mechanism, these reports indicate that COVID-19 infection affects retinal vasculature. A previous report described a case of occlusive retinal vasculitis with H1N1 influenza A, but it is apparently very rare [76].

### 2.3. Clinical Trials of Treatments for Retinal Artery Occlusion

There are no established treatments for retinal artery occlusion so far, even though numerous studies have been conducted to identify a therapy with beneficial effects against visual impairment. It is still unclear whether classical treatments, such as ocular massage, anterior chamber paracentesis to lower the retinal artery perfusion pressure, and hyperbaric oxygen therapy, result in significantly beneficial improvement [77,78]. Any of these conservative therapies may have been proved to be futile in comparison with the natural history [35].

Surgical interventions, including neodymium-doped yttrium aluminum garnet (Nd:YAG) laser embolysis and thrombolysis therapy using tPA (tissue-type plasminogen activator), are still controversial because they do not have established evidence, and there are several risks of hemorrhagic complications [79,80]. Besides, fibrinolytic agents cannot dissolve cholesterol nor calcified material [9]. However, thrombolytics by tPA (intra-arterially by direct administration into the ophthalmic artery or intravenously) is widely administrated for the purpose of recanalization of the retina. The intra-arterial administration of tPA may have marked benefits on visual improvement with less risk of systemic complications [81,82]. Given the fact that there is no inflow from the ophthalmic artery into central retinal artery during CRAO, intra-arterial tPA is more efficient than intravenous administration. However, intra-arterial administration may not be recommended, because there were no differences in visual improvement between intra-arterial tPA and classical treatment in a multicenter trial [83]. Besides, it may cause cerebral and cerebellar hemorrhage. Intravenous tPA is safer and administration within 4.5 h after onset can be beneficial [84]. Apparently, it is not effective at least longer than 6 h after the occurrence [85]. In other words, a prompt diagnosis and quick set-up for thrombolysis are essential for treatment of CRAO. Therefore, only few patients with CRAO receive intravenous thrombolysis appropriately [86].

## 3. Retinal Vein Occlusion

### 3.1. Etiology of Retinal Vein Occlusion

Retinal vein occlusion includes central retinal vein occlusion (CRVO) and branch retinal vein occlusion (BRVO). The prevalence of CRVO is 0.1 to 0.2%. BRVO is more common than CRVO and the prevalence of BRVO is 0.5 to 2.0% [87]. The incidence of BRVO is reportedly 0.5% to 1.2% [88]. In this study, 49% of patients with CRVO are at an age of 65 years or younger and 16% are aged 45 years old or under [89]. The risk of retinal vein occlusion increases with age [90]. Epidemiologically, hypertension is the strongest risk factor for retinal vein occlusion [91]. Diabetes, hyperlipidemia, and hyperuricemia have been revealed as systemic risk factors [92,93,94]. Thrombophilia is an important risk factor of retinal vein occlusion [95]. It is particularly associated with retinal vein occlusion among young patients [96]. Ocular risk factors are glaucoma, ocular hypertension, hypermetropia, and shorter axial length [97,98,99,100]. Pathologically, the arteriovenous crossing site is the origin of BRVO, where the retinal artery crosses over the retinal vein and both exist in a shared adventitial sheath [101,102,103]. The sclerotized artery, with high resistivity, compresses the retinal vein [104,105], which leads to the blockage of blood flow from the affected vein. The occlusion site in the majority of CRVO is within the optic nerve posterior to the lamina cribrosa [9,14].

Clinically, eyes with retinal vein occlusion exhibit flame-shaped and/or dot-blot retinal hemorrhages, retinal edema, tortuous vessels, macular ischemia, and retinal nonperfusion areas [106]. The primary cause of visual impairment is the fact that retinal vein occlusion is macular edema and ischemia, which causes disorganization of the retinal inner layers [107,108]. Visual impairment because of BRVO depends on whether it involves the foveal zone. The initial visual acuity in temporal BRVO was 20/60 or better in 51% and 20/70 or worse in 49% [14]. The prognosis for CRVO is generally worse than BRVO. A visual acuity of 20/200 or worse at the initial visit was observed in 99% of ischemic CRVO and 22% in nonischemic CRVO [14]. The incidence of the conversion of nonischemic CRVO into ischemic CRVO is 13.2% in six months and 18.6% in 18 months [89]. Functional rests such as relative afferent pupillary defect and electroretinography are more useful to differentiate ischemic from nonischemic CRVO than fluorescein angiography [9]. CRVO can be devastating when complications with neovascular response occur. Chronic ischemia in a large area of the retina caused by CRVO and hemi-CRVO, ultimately, induces retinal neovascularization, which causes intravitreal hemorrhage, traction retinal detachment, and neovascular glaucoma. Neovascular glaucoma develops in 36% patients with ischemic CRVO [109]. Cryotherapy is utilized to suppress iris neovascularization and neovascular glaucoma [110].

### 3.2. Treatment Strategy for Retinal Vein Occlusion

While the management of retinal artery occlusion mainly focuses on a systemic assessment, as described above, retinal vein occlusion does not usually need a systemic approach. Therapeutics against retinal vein occlusion have been established thus far. Vascular endothelial growth factor (VEGF) is a crucial driver of retinal edema and hypoxic response during ischemia in retinal vein occlusion as well as nAMD and diabetic retinopathy [111]. Therefore, anti-VEGF therapy, such as bevacizumab, ranibizumab, and aflibercept, is most commonly used against complications secondary to retinal vein occlusion. Large multicenter trials have been performed to evaluate the therapeutic effects of anti-VEGF drugs, which showed a significant improvement in both visual acuity and CST [112,113,114,115,116,117,118]. Anti-VEGF therapy also reduced the retinal nonperfusion area [119,120], which prevented neovascular complications [115]. Among the anti-VEGF drugs, there were nonobvious differences in potency. In the SCORE2 study, bevacizumab had an equivalent effect to aflibercept against visual impairment and macular edema secondary to CRVO and hemi-CRVO [121]. The LEAVO study demonstrated that aflibercept and bevacizumab were noninferior to ranibizumab in CRVO patients [122,123]. As systemic antiplatelet agent and anticoagulant, including aspirin, did not improve visual and morphological outcomes, these treatments apparently do not have preventive nor beneficial effects against retinal vein occlusion [9,124].

Although the therapeutic effects of anti-VEGF therapy for retinal vein occlusion have been elucidated, and have contributed tremendously to visual improvement so far, the optimal treatment approach and strategy may not yet have been established. Frequent intravitreal injections are still heavy burdens, and this makes it difficult for patients to maintain periodic visits and treatment over the long term. Therefore, the development of a protocol with a smaller number of injections but equivalent effects is in demand.

For the management of retinal vein occlusion, a fixed, pro re nata (PRN), or treat and extend (TAE) regimen is used among retinal specialists. Regardless of the treatment protocol, the treatment frequency in the first year affects the visual outcome [125]. In CRVO, the number of injections with PRN regimen in the first year is approximately eight times [126]. Patients with BRVO are treated for approximately seven times within the first year [127]. Inadequate treatment may lead to the limited effects. Besides, considering that frequent visits are necessary for the treatment and approximately half of patients with retinal vein occlusion need the treatment in a long term [128], it may be essential to find other protocols for better and efficient management. TAE regimen is a protocol originally designed for nAMD [125,129,130]. In the TAE regimen, three consecutive injections every 4 weeks are performed as an initial dosing. Afterward, the treatment interval can be extended by 2 weeks if ophthalmological examination, including visual acuity and optical coherence tomography, determine the condition to be stable. In the case that there is any evidence of disease activity, the treatment interval is reduced by 2 weeks to achieve stability [130,131]. The TAE regimen is also applicable to retinal vein occlusion and diabetic macular edema, which have been evaluated in previous studies [132,133,134,135,136,137]. In the CENTERA study, which used a TAE regimen of aflibercept, the mean change in the visual acuity of patients with CRVO was 19.9 letters and their number of treatments was approximately 9.2 over 52 weeks [133]. Another study showed a mean visual improvement in CRVO of 14.8 letters in 12 months and the mean number of injections was 8.3 with a treatment interval of 7.0 weeks [138], suggesting the effectiveness of TAE and the potential for reducing the number of injections. In comparison with monthly injections, CRVO or hemi-CRVO patients with good treatment responses can reduce the number of injections by shifting from monthly injections to a TAE regimen of aflibercept (2.0 times in 6 months) [139]. Among anti-VEGF drugs, the number of injections with TAE regimens using aflibercept for CRVO is reportedly fewer than that using ranibizumab [132].

Some studies modified the TAE regimen with longer intervals (i.e., 4 weeks). The PLATON study utilized a TAE regimen using aflibercept with an interval of 4 weeks. In this study, the mean visual function of BRVO improved by 23.6 letters at 52 weeks [127], and the effect on visual improvement was comparable to 6 monthly injections followed by a bimonthly injection [140]. Arai et al. reported a modified TAE regimen with a 4-week interval [141]. They did not employ initial dosing in order to observe the duration of the recurrence of macular edema after the first injection and to prevent overtreatment. Visual acuity improved by approximately 13 letters [142], which was similar to other protocols [140,142,143,144]. Furthermore, their modified TAE regimen with a 4-week interval maintained visual acuity and dry macula, even after 2 years [145]. As there is still a heavy treatment burden of repeated injections for both patients and healthcare providers, these studies provide important findings for the further improvement of the protocol for anti-VEGF therapy.

### 3.3. Clinical Trials of New Treatments for Retinal Vein Occlusion

As the next therapeutic strategies, several studies have been conducted to determined better and longer-acting therapies [146]. Faricimab is a bispecific antibody that binds and inhibits VEGF-A and angiopoietin-2 [147]. As phase III, multicenter trials for BRVO and CRVO are ongoing (Table 2) [148,149], faricimab may be utilized as an option for long-acting therapy soon.

A certain number of patients with retinal vein occlusion show resistance to anti-VEGF therapy [24]. Therefore, several clinical studies and animal experiments have been conducted to find new approaches and potential targets [150,151]. Anti-VEGF-resistant patients may be improved by an intravitreal dexamethasone (DEX) implant. An intravitreal DEX implant is a rod-shaped implant made of a solid biodegradable polymer, which allows for sustained release of a corticosteroid over a period of 180 days [152]. Ozurdex^®^ (Allergan Inc., Irvine, CA, USA) is a DEX intravitreal implant utilized for diabetic macular edema, retinal vein occlusion, and posterior segment uveitis. Visual acuity and CST in CRVO resistant to anti-VEGF therapy can be improved by a DEX implant [153]. A sham-control clinical trial (i.e., GENEVA study) revealed a significant improvement in visual acuity and a reduction in CST from days 30 to 90 by DEX implant in patients with CRVO and BRVO [154]. Another sham control trial showed a significant improvement in visual acuity at 90 days after treatment for persistent macular edema owing to the fact of retinal vein occlusion, diabetes, uveitis, or Irvine–Gass syndrome, which indicates that a DEX implant is a potentially long-acting therapy [155]. In comparison with anti-VEGF therapy, single administration of a DEX implant was noninferior to ranibizumab by PRN regimen for CRVO in change in visual acuity and CST at months 1 and 2. However, the visual acuity of patients treated with a DEX implant declined at 3 months [156]. A similar tendency was observed in patients with BRVO [157], indicating that macular edema recurs at 3 to 6 months after a single DEX implant. Bandello et al. employed a DEX implant at day 1 and month 5, with the option of retreatment at month 10 or 11 for BRVO. However, in this study, the DEX implant could not show noninferiority to ranibizumab at 12 months [158]. These trials indicate that the superiority of a DEX implant over anti-VEGF therapy is controversial. Furthermore, one of the biggest concerns is the incidence of adverse events such as cataract formation and the elevation of intraocular pressure [159]. Therefore, a DEX implant may be considered as a second-line treatment for patients with a poor response to anti-VEGF injections [154,160,161].

Rho-associated kinase (ROCK) inhibitor also has the potential to suppress retinal neovascularization and macular edema. A previous report using an animal model demonstrated that ripasudil, a ROCK inhibitor, suppressed retinal edema and retinal ischemia in murine retinal vein occlusion [162]. The combination of bevacizumab and another ROCK inhibitor, fasudil, enhanced visual improvement and prolonged its beneficial effects against diabetic macular edema over the long term compared with bevacizumab monotherapy [163], suggesting that rho/ROCK signaling can be a therapeutic target for retinal vein occlusion as well as glaucoma.

A poor response to anti-VEGF therapy may be ameliorated by maintaining a certain concentration of anti-VEGF drugs in the vitreous cavity. Ranibizumab port delivery system (RPDS) has significant potential as a next therapeutic option. It can deliver ranibizumab continuously into the vitreous for 6 months and beyond [164]. Patients with nAMD treated by an RPDS with fixed 24-week refill-exchanges showed noninferiority to intravitreal injection of ranibizumab every 4 weeks [165]. A port delivery system would be a better option to lighten the treatment burden, as almost all patients treated with RPDS preferred the treatment to the classical protocol by intravitreal injection [166]. KSI-301 is a new intravitreal anti-vascular endothelial growth factor (VEGF) antibody biopolymer conjugate. KSI-301 has strong affinity for VEGF-A. It contains high molecular weight phosphorylcholine biopolymer by which the intraocular durability and the duration of the pharmacological effects increase. Consequently, it enhances the beneficial effects in the long term [167]. Phase III trials for retinal vein occlusion as well as nAMD and diabetic macular edema are ongoing (Table 2) [168]. These trials will provide the possibility of KSI-301 for long-acting therapy against VEGF-driven retinal diseases.

## 4. Conclusions and Future Perspectives

Retinal occlusive diseases are serious disorders that clinicians commonly see in daily medical practice. Although the current therapies and protocols developed have contributed to improvements in the quality of life and vision, research on better management will be performed as well as studies to determine new therapeutic options such as gene therapy, cell therapy, and some new molecular targets over the next years and decade. Further clinical interventions and animal studies will be necessary for understanding the detailed pathogenesis and to explore new therapeutic avenues.

## Figures and Tables

**Table 2:** Ongoing Phase I–IV clinical trials for retinal vein occlusion.

NCT Number	Phase	Conditions	Treatments	Purpose
NCT03981549	1/2	CRVO	Autologous bone marrow CD34+ stem cells	Evaluating the safety and feasibility of intravitreal injection of autologous bone marrow CD34+ stem cells.
NCT04707625	4	RVO	Aflibercept	Measuring the levels of VEGF in aqueous humor to predict the timing of retreatment.
NCT03709745	4	BRVO	Aflibercept, ranibizumab	Comparing the time to the first recurrence of macular edema after an initial loading dose.
NCT05133791	1	RVO	Annexin A5-CW800	Near-infrared fluorescent imaging in the retina of patients with RVO related to the systemic injection of annexin A5-CW800.
NCT04444492	3	CRVO	Ranibizumab, laser photocoagulation	Evaluating the long-term effect of laser photocoagulation in combination with ranibizumab.
NCT04592419	3	RVO	KSI-301	Evaluating the efficacy and safety of intravitreal KSI-301.
NCT04740905	3	BRVO	Faricimab	Evaluating the efficacy, safety, and pharmacokinetics of faricimab.
NCT05282420	4	CRVO	Aflibercept, ranibizumab	Comparing the efficacy and safety of ranibizumab and aflibercept for CRVO (younger than 50 years old).
NCT04576689	2	RVO, DME	IBE-814	Evaluating the comparative safety and preliminary efficacy of IBE-814 IVT (dexamethasone implant).
NCT05290948	2	RVO	Bevacizumab with oral acetazolamide tablets	Comparison of combined intravitreal bevacizumab and oral acetazolamide versus intravitreal bevacizumab alone.
NCT04740931	3	CRVO, hemi-CRVO	Faricimab	Evaluating the efficacy, safety, and pharmacokinetics of faricimab.
NCT04563299	4	RVO, DME, nAMD	Dextenza	Evaluating pain and inflammation with DEXTENZA treatment.
NCT03056079	4	RVO, DME, nAMD	Aflibercept	Investigating the association between cytokine levels in aqueous humor and the optimal treatment interval.
NCT03056092	4	RVO, DME, nAMD	Ranibizumab	Investigating the association between cytokine levels in aqueous humor and the optimal treatment interval.
NCT05112861	3	BRVO, DME, nAMD	Bevacizumab	Comparing the safety of ONS-5010 in vials and prefilled syringes.
NCT04120636	1	BRVO, ERM, RR, CSC with pit of optic disc, vitritis, commotio retinae	Episcleral celecoxib	Assessing the safety, tolerability, and pharmacokinetics of episcleral celecoxib in patients with macular edema and other inflammatory disorders of the retina, choroid, and vitreous.

Accessed at “http://www.clinicaltrials.gov” on 16 September 2022. Searched using “retinal vein occlusion”. Recruitment status was “active, not recruiting” or “recruiting”. Study phases from I to IV were included. RVO: retinal vein occlusion; DME: diabetic macular edema; nAMD: neovascular age-related macular degeneration; RR: radiation retinopathy; ERM: epiretinal membrane; CSC: central serous retinopathy.
